# Novel SA@Ca^2+^/RCSPs core–shell structure nanofibers by electrospinning for wound dressings†

**DOI:** 10.1039/c8ra00784e

**Published:** 2018-04-24

**Authors:** Rui Li, Zhiqiang Cheng, Ruicheng Wen, Xiaodong Zhao, Xiaobin Yu, Lin Sun, Yingying Zhang, Zhiyuan Han, Yafeng Yuan, Lijuan Kang

**Affiliations:** College of Resources and Environment, Jilin Agriculture University Changchun 130118 People's Republic of China czq5974@163.com; College of Life Sciences, Jilin Agricultural University Changchun 130118 People's Republic of China; College of Aerospace Engineering, Tsinghua University Beijing 100000 People's Republic of China

## Abstract

Therapeutic drugs remain of great significance for the absorption of wound blood, the closure of wounds and rapid wound healing. Hence, we propose a novel composite nanofiber membrane with the above characteristics as a wound healing material. We utilize the reaction of calcium ion and alginate gel, sodium alginate (SA) and *Rana chensinensis* skin peptides (RCSPs) extracted from discarded *Rana chensinensis* skin; these two natural substances were successfully used to prepare composite nanofibers by coaxial electrospinning. The composite nanofibers are named SA@Ca^2+^/RCSPs nanofibers. SA@Ca^2+^/RCSPs nanofibers exhibited that the nanofibers contact with the liquid is unmelted, instead become gel, when compared to nanofibers of does not contain calcium ions, and the absorption rate reached 179.87%. SA@Ca^2+^/RCSPs nanofibers conform to the quasi-first-order dynamics model and the Ritger–Peppas release model. *In vivo* wound healing experiments showed that the wound-healing rate of SA@Ca^2+^/RCSPs nanofiber-treated wounds was 46.65% and 97.46% on days 5 and 15, respectively. In addition, SA@Ca^2+^/RCSPs nanofibers promoted collagen deposition and enhanced epidermal regeneration. The present study showed that composite nanofibers could quickly undergo hemostasis and effectively promote wound healing.

## Abstract 2

Core–shell nanofiber membranes by coaxial electrospinning process that became gel after absorbing blood were used for wound repair.

## Graphical abstract

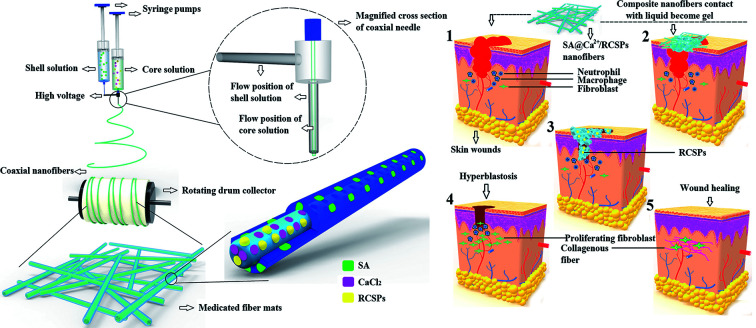

## Introduction

In daily life, skin trauma is unavoidable because of various causes. The wound healing process includes hemostasis, inflammation, cell proliferation and tissue reconstruction.^1^ To facilitate the treatment of wounds, an increasing number of patients use multi-functional composite wound dressings. After the wound dressing is applied to the wound site, wound bleeding can rapidly be stopped, and the wound organization exhibits a certain prosthetic effect. Ideal wound dressings should maintain proper moisture,^2,3^ allow gas exchange, absorb excess exudates and maintain a damp environment. These conditions are beneficial for the generation of a hypoxic environment, promote capillary formation, enhance epithelial growth, avoid palingenetic epithelial tissue with dressing adhesion and reduce pain.^4–8^

Alginate is extracted from natural brown seaweed. Alginate has good biocompatibility,^9^ and exhibits non-toxic,^10^ safe and mild gelation by the addition of divalent cations, such as Ca^2+^.^11,12^ Alginate can be used to deliver a variety of drugs.^13,14^ Alginate dressing has been used in post-operative wounds, ulcers, *etc.* and is conducive for wound healing, skin regeneration and reducing pain. Saarai *et al.*^15^ prepared gel by mixing alginate with gelatin, which exhibit high absorption for liquid. Alginate is widely used in biomedical applications.^16,17^ Alginate can optimize the performance of wound dressings, and thereby accelerate wound healing.

*Rana chensinensis* is a medicinal and edible amphibian economy animal, widely distributed in northern China. RCSPs are extracted from discarded *Rana chensinensis* skin, and the molecular weight of these compounds is 3.5 kDa. RCSPs mainly consists of peptides and proteins.^18^ Studies on RCSPs for the effect of proliferation of mouse fibroblasts have shown that these polypeptides have low toxicity towards normal cells.^19^ Additionally, RCSPs exhibit good biocompatibility^20^ and absorbability.^21,22^ A novel peptide from skin secretions of the odorous frog *Odorrana margaretae* can promote the formation of keratinocytes and human skin fibroblasts.^23^ Collagen peptide is widely used in wound healing, as this protein promotes wound healing and accelerates the healing speed.^24,25^

Currently, nanocomposites are widely used.^26–29^ Because the composite material maintains the advantages of the performance of each individual material and also the complementarity and correlation of the performances of the materials together, better overall performance can be achieved.^30,31^ For example, changing the performance of the original material,^32,33^ increased compatibility^34^ lipophilicity,^35^*etc.* Electrostatic spinning technology is particularly suitable for the preparation of biological materials,^36–39^ which can control drug release over time.^40–44^ The nanofibers prepared by electrospinning have several advantages, such as high porosity and large specific surface area.^45^ The technology is simple and low cost, with strong generality.^46,47^ Co-axial electrospinning can separate core solutions from shell solutions, avoiding contact between the two solutions until the last moment when they exit the nozzle.^48^

In the present study, we prepared SA@Ca^2+^/RCSPs composite nanofibers by coaxial electrospinning using polyvinylpyrrolidone (PVP). PVP is a macromolecule compound widely used in the biological field. PVP has been used as a blood plasma substitute.^49^ PVP has been used as a rapidly dissolving matrix carrier in several drug delivery system formulations.^50^ The necessity of coaxial electrospinning was studied to analyze the fiber morphology and contact angle. The adsorption kinetics and drug release kinetics of SA@Ca^2+^/RCSPs composite nanofibers were studied by measuring their adsorption quantity and drug release properties, respectively. The full thickness excisional wound model was used to assess the wound healing efficiency of nanofibers with RCSPs *in vivo*. Compared with previous reported studies, we not only easily cross-linked nanofibers but also generated a moist environment and accelerated wound healing. The purpose of the present study was to develop dressings for wound hemostasis, the rapid release of drug and acceleration of wound healing.

## Experimental section

### Materials

*Rana chensinensis* skin peptides (RCSPs) were obtained from Fang Ping Technology Co., Ltd. (Jilin, China). Sodium alginate (SA), calcium chloride and sodium chloride were purchased from Shanghai Chemical Reagent Purchasing Supply Station (Shanghai, China). Polyvinylpyrrolidone (PVP, *M*_w_ = 630 000) were purchased from Ourchem Chemical and Technology Co., Ltd (Guangzhou, China). Phosphate buffered saline (PBS) was purchased from Haibiao Chemical and Technology Co., Ltd (Xiamen, China). Deionized water is provided in our lab.

### Preparation of coaxial electrospinning solution

PVP were dissolved in deionized water to prepare two concentrations of solutions; a and b solutions were 8% (w/v) and 11% (w/v), respectively. Calcium chloride was dissolved in deionized water to obtain 2% (w/v) calcium chloride solution. The appropriate amount of RCSPs dissolved in solution a. The solutions were stirred at room temperature for 2 hours, and then the appropriate amount of calcium chloride solution was added to the solutions, followed by stirring at room temperature for 2 h. The mixed solution was named A. Sodium alginate was dissolved in deionized water to obtain 2% (w/v) sodium alginate solution. Sodium alginate solution was mixed with solution b (2 : 8), and the mixed solution was named B, which was stirred at room temperature for 4 hours.

### Electrospinning of coaxial nanofibers

Coaxial electrospinning experiments were performed at room temperature. Solution A is the core solution and solution B is the shell solution.^51^ Solution A was loaded in a 5 ml syringe connected to an internal, and solution B was loaded in 5 ml syringe connected to an external needle. The ratio of the flow rates of core and shell solutions was 1 : 3.^43^ The applied voltage was 20 kV, and the distance from the tip to the collector was 15 cm. According to the above steps, for the calcium chloride solution not added to solution A, the coaxial nanofibers were named SA@RCSPs nanofibers. All nanofiber mats were dried under vacuum at room temperature for 5 days.

### Characterization of SA@Ca^2+^/RCSPs and SA@RCSPs nanofibers

The morphologies of nanofibers were observed using scanning electron microscopy (SEM, SHIMADZU S-550, operating voltage: 15 kV). The core–shell structure of the coaxial nanofibers was observed using transmission electron microscopy (TEM, Tecnai G2, operating voltage: 200 kV, exposure time is auto exposure and time is 2 seconds). The pore size of the nanofibers was measured using the membrane pore size analyzer (3H-2000PB, Beishide Instrument Technology (Beijing) Co., Ltd). The crystalline nature of the nanofibers was analysed by X-ray photoelectron spectroscopy (XPS, SHIMADZU, scan speed: 2 deg min^−1^). The thermal properties of nanofibers and RCSPs were studied using thermogravimetric (TG, HCT-3, Beijing HengJiu Scientific Instrument Factory, heating rate: 10 °C min^−1^) and differential scanning calorimeter (DSC, HSC-3, Beijing HengJiu Scientific Instrument Factory, heating rate: 10 °C min^−1^). The Fourier transform infrared spectrometer (FTIR-650, TianJin GangDong Sci. & Tech. Development Co., Ltd) was used to obtain the ATR-FTIR spectra of RCSPs, SA nanofibers, SA@RCSPs nanofibers and SA@Ca^2+^/RCSPs nanofibers in the region of 400–4000 cm^−1^. Liquid infiltration was measured for SA@Ca^2+^/RCSPs nanofibers and SA@RCSPs nanofibers using optical contact angle & interface tension meter (SL200KS, USA KINO Industry Co., Ltd).

### Formed gel of test

The coaxial nanofiber can be melted by water due to its strong hydrophilicity. To improve the stability and unchangeable chemical structure of the RCSPs in the nanofibers, we used an ion cross-linking reaction between sodium alginate and calcium ions, which leads to a gel structure. Using a syringe, calcium chloride solutions were dropped into a Petri dish containing a mixed solution of solution b with sodium alginate solution. NaCl–CaCl_2_ solution was prepared by dissolving 4.149 g of NaCl and 0.184 g of CaCl_2_ in a 500 ml volumetric flask with deionized water (according to YYT0471.1-2004, contact wound dressing test method); the NaCl–CaCl_2_ solution was named M. The ion content of the solution was equivalent to human serum albumin or wound exudate. Solution M was loaded into a syringe, then the appropriate amount was dripped onto the prepared SA@Ca^2+^/RCSPs nanofiber mats, and the results were observed.

### Liquid absorption performance test

The ratio of the absorption liquid of SA@Ca^2+^/RCSPs nanofibers was assessed by adsorption assay for solution M. At room temperature, SA@Ca^2+^/RCSPs fiber mats were cut into 5 × 5 cm^2^ then the mass of the fiber mats was accurately weighed. The fiber mats were immersed in solution M at 37 ± 1 °C for a different time until the fiber mats adsorption was saturated. After imbibition, surplus solution in the fiber mats were filtered. The liquid absorption rate was calculated using the following formula.
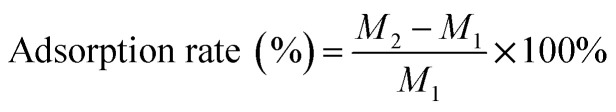
*M*_1_ is the quality of the dry fiber mats and *M*_2_ is the quality of the fiber mats after imbibition.

### *In vitro* release studies

The release profile of RCSPs from SA@Ca^2+^/RCSPs nanofiber matrix was investigated in phosphate-buffered saline (PBS) at 37 ± 1 °C. Standards were prepared by dissolving known amount of RCSPs in solvent. Standard graphs were plotted using absorbance at 540 nm which is specific for peptide linkage. The SA@Ca^2+^/RCSPs fiber mats (5 mg) were cultured in 3 ml of PBS at 37 ± 1 °C for 10 min. At specific time intervals, 1 ml of solution was withdrawn from the release medium, and the same volume was replaced with fresh PBS. The relative concentration of the RCSPs was determined using an ultraviolet and visible spectrophotometer (T6 new century, Beijing Purkinje General Instrument Co., Ltd) at a wavelength of 540 nm by comparison with the standard plot.

### *In vivo* wound healing experiments

Healthy male black mice (C57) weighing 17–20 g were used for wound healing experiments. All animal procedures were performed in accordance with the Guidelines for Care and Use of Laboratory Animals of Jilin Agricultural University and experiments were approved by the Animal Ethics Committee of Jilin Agricultural University. The mice were maintained on a normal diet and drinking water. Altogether, 24 black mice were divided into four groups. The black mice were anesthetized by an enterocoelia injection with 4% chloral hydrate (0.01 ml g^−1^). The dorsal surface of the black mice was shaved, then disinfected with 70% ethanol and a full thickness (diameter of 1 cm round) excision wound was created by excising the dorsal skin. Group 1 (control) animals were treated with normal saline, group 2 animals were treated with PVP nanofibers, group 3 animals were treated with SA@Ca^2+^ nanofibers and group 4 animals were treated with SA@Ca^2+^/RCSPs nanofibers. The wounds were wiped with normal saline before changing the dressing materials daily. The percentage of wound closure was calculated by the following formula.
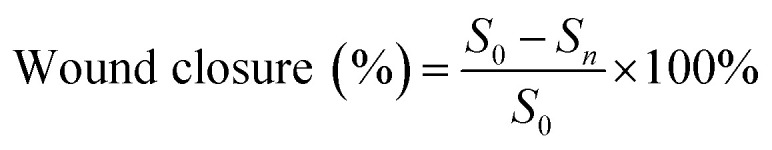
*S*_0_ is area of original wound and *S*_n_ is area of actual wound (on days 5, 10, and 15 post wounding).

The black mice from each group were euthanized on days 7 and 15 post-treatment for histological analysis.

### Histological analysis

Wound tissues on post-operative days 7 and 15 were maintained in cold 4% paraformaldehyde in 0.01 M PBS (pH = 7.4) overnight and embedded in paraffin. The paraffin samples were cut into slices with a thickness of 5 μm using a Paraffin slicing machine (Leica RM2235) for staining with Hematoxylin–Eosin (H&E) and Masson's trichrome staining. Histological sections were observed using a Zeiss Axio Scope Microscope.

### Statistical analysis

All data were expressed as average ± standard deviations. Statistical differences were analyzed using one-way analysis of variance (ANOVA) with GraphPad Prism 6 software. For all tests, *p value < 0.05, **p value < 0.01, and ***p value < 0.001.

## Results and discussion

### Morphology and characterizations of electrospun nanofibers

**Fig. 1:**
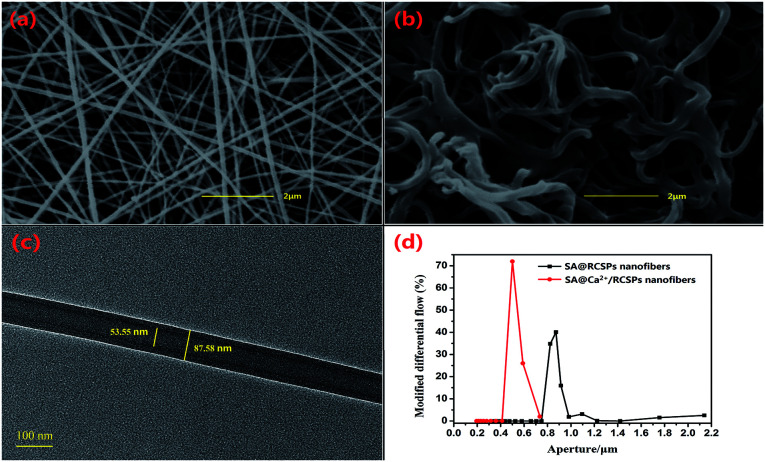
(a) SEM images of SA@RCSPs nanofibers, (b) SEM images of SA@Ca^2+^/RCSPs nanofibers, (c) TEM images of SA@RCSPs nanofibers, (d) aperture distribution diagram of SA@Ca^2+^/RCSPs nanofibers and SA@RCSPs nanofibers.

Coaxial nanofibers were successfully prepared by coaxial electrospinning.^48^ As the shell solution has a large viscosity, the viscous stress of the inner solution was sufficient to overcome the internal tension between the two solutions, the shell has a sufficient surface tension to balance the electric field force under equilibrium forces, and the tip can form stable composite Taylor Cone.^51^ However, the larger viscosity of the shell solution can also guide the core solution to better form nanofibers. SEM images of SA@Ca^2+^/RCSPs nanofibers and SA@RCSPs nanofibers after vacuum drying are shown in Fig. 1 (a and b, respectively). The nanofibers were smooth, uniform and straight that did not contain calcium chloride. Nanofibers containing calcium ions after vacuum drying became bent, which could be ejected from the core–shell solution, and then the sodium alginate of the shell solution and the calcium ions of the core solution could form an ion exchange reaction after the nanofibers absorb moisture, resulting in cross-linking. Fig. 1(c) shows the TEM images of SA@RCSPs nanofibers. The diameter of the shell fiber was 87.58 nm and the diameter of the core fiber was 53.55 nm, indicating that coaxial nanofibers can be obtained by coaxial electrospinning. Fig. 1(d) shows the pore size distribution of SA@Ca^2+^/RCSPs and SA@RCSPs nanofibers. The average pore size of SA@RCSPs nanofibers was 0.9087 μm and the average pore size of SA@Ca^2+^/RCSPs nanofibers was 0.5713 μm due to bending and swelling of the nanofibers after gelatinization, resulting in SA@Ca^2+^/RCSPs nanofibers with smaller pore sizes than SA@RCSPs nanofibers.

**Fig. 2:**
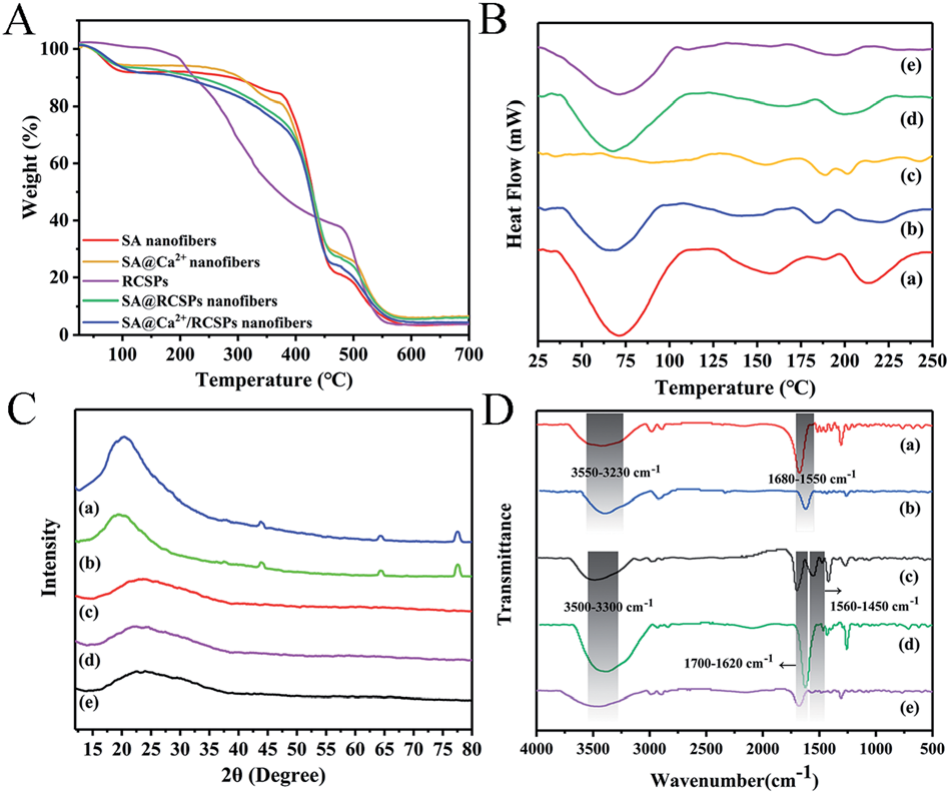
(A) TG analysis for SA nanofibers, SA@Ca^2+^ nanofibers, RCSPs, SA@RCSPs nanofibers and SA@Ca^2+^/RCSPs nanofibers. (B) DSC thermograms of (a) SA nanofibers, (b) SA@Ca^2+^ nanofibers, (c) RCSPs, (d) SA@RCSPs nanofibers, (e) SA@Ca^2+^/RCSPs nanofibers. (C) XRD patterns of (a) SA nanofibers, (b) SA@Ca^2+^ nanofibers, (c) SA@RCSPs nanofibers, (d) SA@Ca^2+^/RCSPs nanofibers, (e) RCSPs. (D) ATR-FTIR spectra of (a) SA nanofibers, (b) SA@Ca^2+^ nanofibers, (c) RCSPs, (d) SA@RCSPs nanofibers, (e) SA@Ca^2+^/RCSPs nanofibers.

TG analysis of SA nanofibers, SA@Ca^2+^ nanofibers, RCSPs, SA@RCSPs nanofibers and SA@Ca^2+^/RCSPs nanofibers is shown in Fig. 2A. The results show that the weight loss curves of SA nanofibers, SA@Ca^2+^ nanofibers, SA@RCSPs nanofibers and SA@Ca^2+^/RCSPs nanofibers are basically the same. The weight loss in the first stage was 8.84%, 6.34%, 7.06% and 9.02% at 40 °C to 110 °C, respectively, due to residual moisture evaporated on the surface and inside of the nanofibers. For SA nanofibers and SA@Ca^2+^ nanofibers, the second stage was from 280 °C to 370 °C (7.71% and 13.61%). The alginic acid was split into intermediate products, and the adjacent hydroxyl groups were removed as water molecules. The third stage was from 370 °C to 460 °C, and the intermediate products are further decomposed and carbonized (62.47% and 51.92%). The fourth stage was from 460 °C to 560 °C, in which carbides of alginic acid were further oxidized and decomposed. For PCSPs, the first stage is from 60 °C to 190 °C, due to the evaporation of water in organics (4.82%). The second stage was from 190 °C to 480 °C because of the carbonization of the organic molecules (59.46%). The third stage was from 480 °C to 550 °C, in which carbides are further oxidate and decomposed (33.30%). For SA@RCSPs nanofibers and SA@Ca^2+^/RCSPs nanofibers, because of the existence of RCSPs, the second stage was from 110 °C to 460 °C (65.71% and 66.77%). The third stage was from 460 °C to 560 °C, in which carbides of RCSPs and alginic acid were further oxidized and decomposed. Obviously, the thermal performance of SA@RCSPs nanofibers and SA@Ca^2+^/RCSPs nanofibers are between SA nanofibers and RCSPs, indicating that the presence of SA increases the thermal stability of the composite nanofibers.

The thermal performance of RCSPs and nanofibers was measured by DSC. Fig. 2B shows the DSC curves of RCSPs and four groups of nanofibers at 25 °C to 250 °C. The first endothermic peak of nanofibers of the four groups was observed at approximately 70 °C, which is the glass transition temperature (*T*_g_), due to residual moisture evaporated on the surface and inside of the nanofibers. The crystallization peak of SA nanofibers occurred at approximately 160 °C. Because the RCSPs composite with SA by coaxial electrospinning, the melting peak of SA@RCSPs nanofibers and SA@Ca^2+^/RCSPs nanofibers are between these molecules (approximately 195 °C), indicating their compatibility.

The X-ray diffraction patterns of RCSPs, SA@RCSPs nanofibers, SA@Ca^2+^/RCSPs nanofibers, SA nanofibers and SA@Ca^2+^ nanofibers are shown in Fig. 2C. SA and SA@Ca^2+^ nanofibers show amorphous peaks appearing at approximately 20.4° and 19.7°, and there were weaker diffraction peaks at approximately 43.9°, 64.4° and 77.6°. Indicating that SA has weaker crystallinity and the main form was amorphous. RCSPs, SA@RCSPs nanofibers, SA@Ca^2+^/RCSPs nanofibers show amorphous peaks at approximately 22.7°, 22.4° and 23.6°. Due to the addition of RCSPs, the crystallization peaks of SA disappeared at 43.9°, 64.4° and 77.6°, indicating that the interaction between RCSPs and alginic acid reduces the crystallinity of the composite nanofibers.

The ATR-FTIR spectra of SA nanofibers and SA@Ca^2+^ nanofibers were presented in Fig. 2D(b and c). The SA nanofibers spectra show O–H stretching vibration and COO– asymmetric stretching at 3422 cm^−1^ and 1676 cm^−1^, respectively. After the addition of calcium chloride, the SA nanofibers show O–H stretching vibration at 3409 cm^−1^ and COO– asymmetric stretching at 1619 cm^−1^ in Fig. 2D(c). These peaks are attributed to the cross-linking reaction of calcium ions. The intermolecular hydrogen bond of sodium alginate was destroyed, so that the O–H stretching vibration moves to a low wave number. The ATR-FTIR spectra of RCSPs (powder), SA@RCSPs nanofibers and SA@Ca^2+^/RCSPs nanofibers are shown in Fig. 2D(a, d and e). The RCSPs characteristic peak at 3490 cm^−1^, 1695 cm^−1^ and 1556 cm^−1^, which corresponds to the –NH stretching vibration, amide I and amide II. The RCSPs nanofibers contain sodium alginate, which causes the vibration frequency of amide I and amide II to shift to 1625 and 1470 cm^−1^, respectively. Primarily because of the electrostatic interaction between alginate and peptide molecules. The spectra of SA@RCSPs nanofibers and SA@Ca^2+^/RCSPs nanofibers showed that the peak of –NH stretching vibration broadened due to the formation of hydrogen bonds between alginate and RCSPs.

**Fig. 3:**
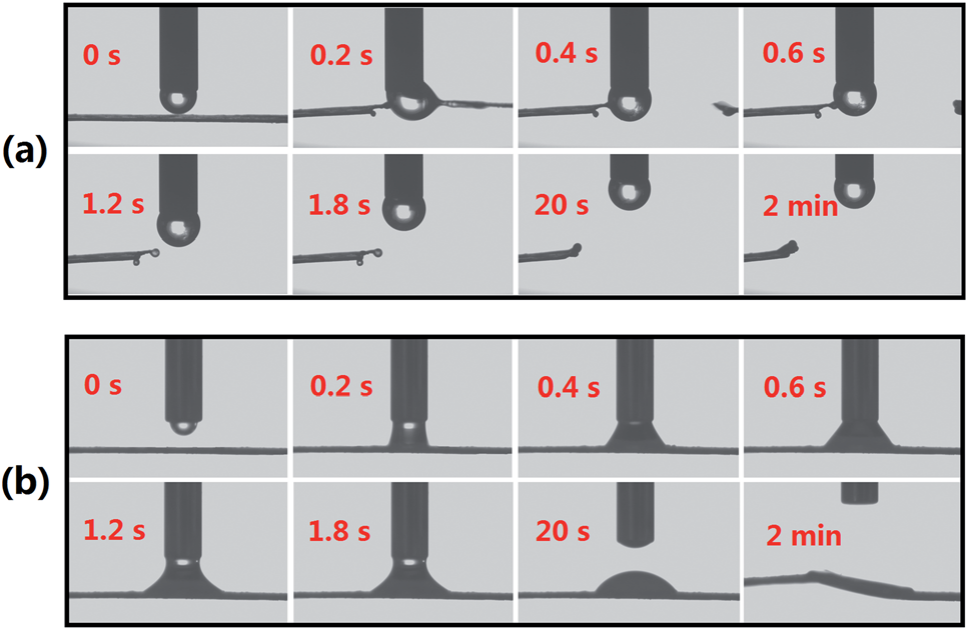
(a) Contact angle images of SA@RCSPs nanofibers, (b) contact angle images of SA@Ca^2+^/RCSPs nanofibers.

SA@RCSPs nanofibers and SA@Ca^2+^/RCSPs nanofibers are given in Fig. 3(a) and (b). Testing liquid in the experiment was solution M. Fig. 3(a) shows that the calcium-free nanofiber mats has strong hydrophilicity. When water droplets contact the nanofiber mats, but have not yet completely dripped onto the mats, the fiber mats began to melt and become cleaved. Fig. 3(b) shows that the water droplets contact the SA@Ca^2+^/RCSPs nanofibers and then the solution was absorbed by the mats because the fiber mats, containing calcium ions, produce gelation reactions. The solution basically infiltrated into the fiber mats after 2 min. The results indicate that SA@Ca^2+^/RCSPs nanofibers were successfully gelled due to the ion exchange reaction of calcium ions with sodium alginate. Solution M was rapidly absorbed after contact with SA@Ca^2+^/RCSPs nanofibers, and the fiber mats were insoluble.

### Gel formation

**Fig. 4:**
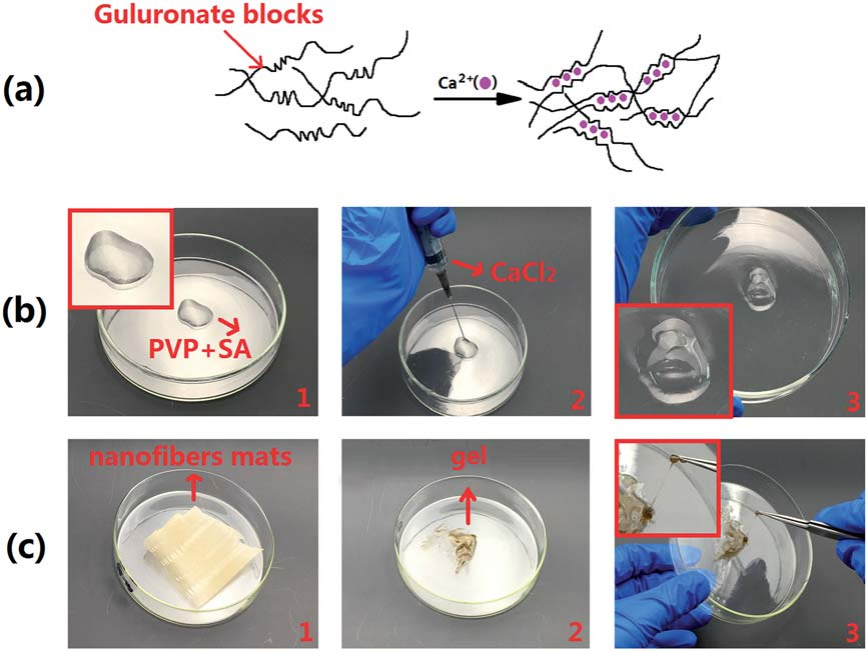
(a) Schematic diagram alginate of hydrogels prepared by calcium ionic cross-linking (egg-box model), (b) calcium chloride solution was added to PVP-SA solution to form gel, (c) SA@Ca^2+^/RCSPs nanofibers formed a gel after absorbing the liquid.

Fig. 4(a) shows that the guluronate blocks of one polymer form junctions with the guluronate blocks of adjacent polymer chains in what is termed the egg-box model of cross-linking under the action of calcium ions, resulting in a gel structure.^52^ The most common method of preparing hydrogels from aqueous alginate solutions was that the solutions combine the ionic cross-linking agents, such as divalent cations (*i.e.*, calcium ion). The divalent cations may bind solely to the guluronate blocks of the alginate chains.^53^ Calcium ions are required in the human body, and these molecules are not toxic to the humans in a certain range, thus becoming the preferred material for alginate gel. Fig. 4(b) shows that immediately after dropping 2% calcium chloride solution into SA-PVP solution, a gel is formed. Therefore, we intend to generate fiber mats that can be green and environmentally cross-linked by using coaxial electrospinning technology. The solution containing calcium ions and the solution containing sodium alginate were separated during the electrospinning process because of the rapid formation of the jet so that the two solutions are not mixed. Fig. 4(c) shows that solution M was dropped onto the fiber mats, and the fiber mats were not dissolved, but formed a gel.

### Liquid absorption performance

**Fig. 5:**
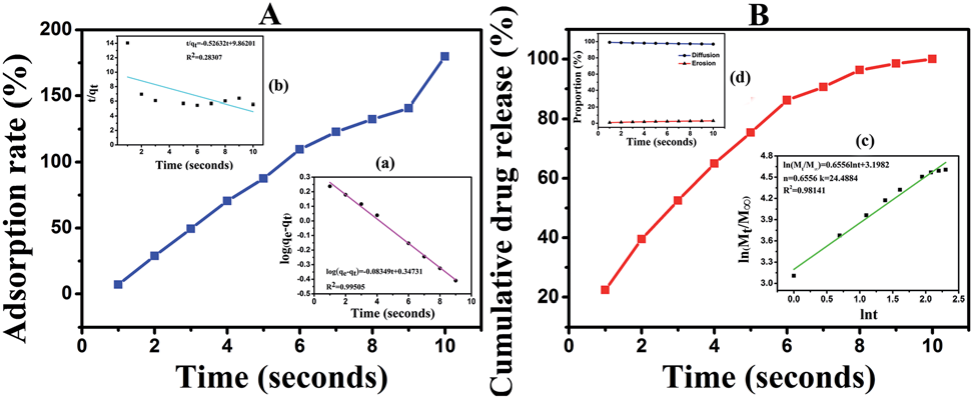
(A) Adsorption rate of SA@Ca^2+^/RCSPs nanofibers for solution M. (a) Quasi-first-order dynamics model. (b) Quasi-second-order dynamics model. (B) Cumulative RCSPs release from SA@Ca^2+^/RCSPs nanofibers. (c) Ritger–Peppas release model. (d) Peppas correction formula.

SA@Ca^2+^/RCSPs nanofibers adsorption solution M is shown in Fig. 5(A) In a short time, the fiber mats quickly absorb solution M and reach saturation in 10 seconds, and eventually reach an adsorption rate of 179.87% thereafter. To discuss the adsorption process, we used the Lagergren quasi-first and quasi-second order kinetic rate equations.

The linear form of the quasi-first order kinetic rate equations is as follows.
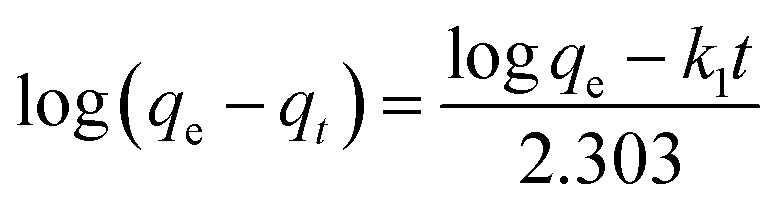


The linear form of the quasi-second order kinetic rate equations is as follows.
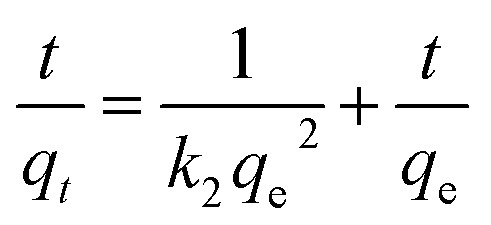
where *q*_e_ and *q*_*t*_ represent the amount of adsorption (g g^−1^) at the adsorption equilibrium and time *t* respectively, and *k*_1_ and *k*_2_ are the adsorption rate constants.

The quasi-first and quasi-second order kinetic rate equations are shown in Fig. 5(a) and (b), respectively, display *R*^2^ = 0.99505, and *R*^2^ = 0.28307. These results show that the adsorption process conforms to the quasi-first-order dynamics model. The quasi-first order kinetic rate equation was log(*q*_e_ − *q*_*t*_) = −0.083491*t* + 0.34731. It is important that quickly enable hemostasis when healing skin wounds.

### *In vitro* release studies

The release of RCSPs from the composite nanofibers is shown in Fig. 5(B). After the nanofibers were immersed in PBS, in a short time, the nanofibers rapidly and completely release the RCSPs. Upon immersion of the nanofibers in PBS, the cumulative release reached 22.38% within the first second. At the tenth second, RCSPs from SA@Ca^2+^/RCSPs nanofibers were completely released. To further understand the diffusion mechanism of RCSPs from the nanofibers, the Ritger–Peppas release model was used to analyze the experimental results by the following formula.^54,55^
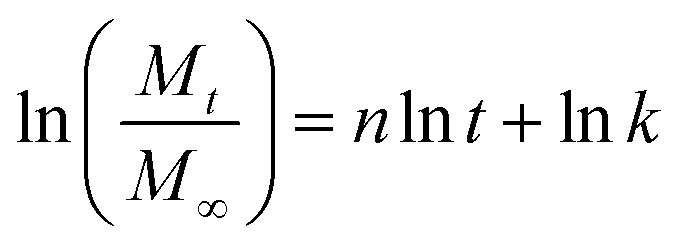
where *M*_*t*_ is the release amount of time *t*, *M*_∞_ is the amount of release at equilibrium, *n* is the diffusion index, *k* is the diffusion constant, and *t* is the release time.

The kinetic parameters *n* and *k* can be calculated to fit the curves, with ln(*M*_*t*_/*M*_∞_) as ordinate and ln *t* as the abscissa curve. As shown in Fig. 5(c), the value of *n* and *k* were 0.6556 and 3.1982, respectively. According to the diffusion mechanism, for the thin film, *n* = 0.5 corresponding to the Fick release, and *n* = 1 corresponds to the case-II transport. When 0.5 < *n* < 1.0, the drug release mechanism was the non-Fick diffusion (Anomalous Transport). Thus, the release of the thin film was the coupling results of two methods (drug diffusion and skeleton dissolution). Because the carrier degradation and dissolution accelerate the drug release rate, the value of *n* is higher than the Fick release.

To quantitatively explain the release of RCSPs from SA@Ca^2+^/RCSPs through the proportion of the drugs diffusion and dissolution, respectively. We used amendment equations proposed by Peppas *et al.* based on the following formula.^55^*W* = *k*_1_*t*^*m*^ + *k*_2_*t*^2*m*^*W* is the cumulative release of RCSPs from the nanofibers, *m* is the Fickian diffusion index, and *k*_1_ and *k*_2_ are the diffusion constants.

The diffusion of the amount of drug release accounting for the proportion of the total release was calculated by the following formula.
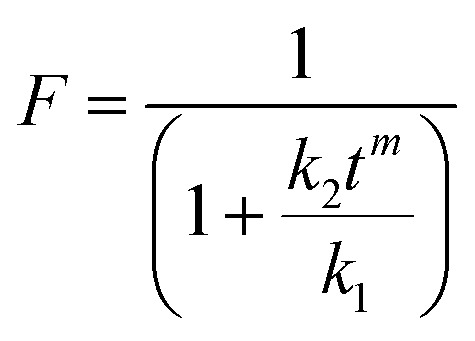


The dissolution mechanism of the amount of drug release accounting for the proportion of the total release was calculated by the following formula.*R* = 1 − *F*

The equation *W* = *k*_1_*t*^*m*^ + *k*_2_*t*^2*m*^ used multiple regression and thus *W* = 28.90711*t*^0.57625^ + 0.24229*t*^1.1525^, *R*^2^ = 0.97724. Using the values of *k*_1_, *k*_2_, the plot for equations *F* = 1/(1 + *k*_2_*t*^*m*^/*k*_1_) and *R* = 1 − *F* to obtain curve of proportions of diffusion release and dissolution release in the amount of accumulated release RCSPs at some point, as shown in Fig. 5(d). The release form of the RCSPs was mainly diffusion. Due to the fiber mats contacting solution M, which quickly became a gel, the drug was immediately diffused. While RCSPs have good water solubility, leading to the gel layer of inner has a higher concentration gradient than the outer. With increasing time, the dissolution mechanism of the amount of drug release accounted for the proportion of the total release that was slightly increased, compared with the diffusion mechanism. Due to inside and outside of the gel layer formation, the concentration gradient gradually became low with increasing time, resulting in a decrease of diffusion release and an increase in dissolved release. During the healing of the wound, the rapid release of drugs and the dissolution of the skeleton of the drugs were desired results.

### *In vivo* wound healing activity

**Fig. 6:**
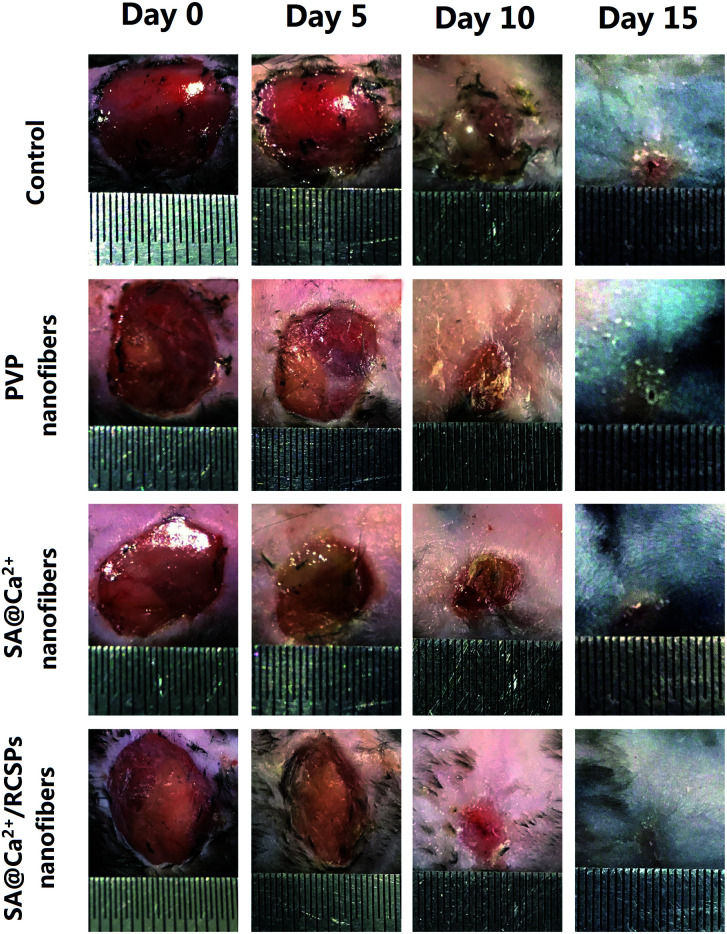
Photograph of the wound healing after treatment with control, PVP nanofibers, SA nanofibers (contain calcium chloride) and SA@Ca^2+^/RCSPs nanofibers on days 5, 10 and 15.

**Fig. 7:**
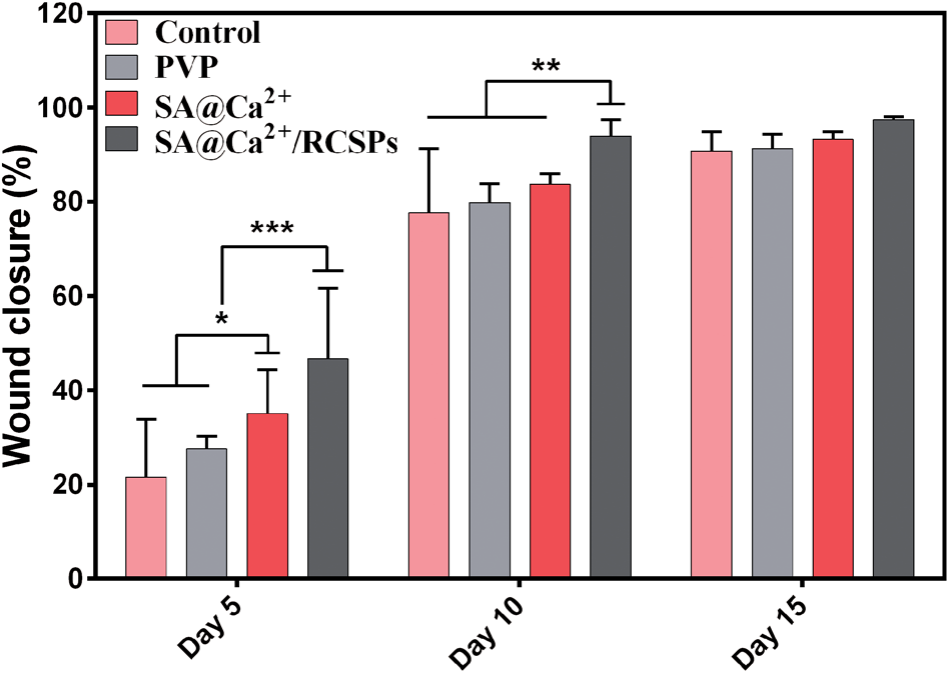
Closure rate of wounds treated with control, PVP nanofibers, SA@Ca^2+^ nanofibers and SA@Ca^2+^/RCSPs nanofibers on days 5, 10 and 15. (*p ≤ 0.05, **p ≤ 0.01, ***p ≤ 0.001).

For wound healing, the experiment was divided into four groups: control, PVP nanofibers, SA@Ca^2+^ nanofibers, SA@Ca^2+^/RCSPs nanofibers (the video† shows that SA@Ca^2+^/RCSPs nanofibers were wiped onto the wound of the black mouse). As shown in Fig. 6 and 7, SA@Ca^2+^/RCSPs nanofibers had the highest wound-healing rate. On the fifth day, the healing rate of SA@Ca^2+^/RCSPs nanofibers was approximately 46.65% whereas the wound healing rates of control, PVP nanofibers and SA@Ca^2+^ nanofibers exhibit healing rates of 21.63%, 27.62% and 35.03%, respectively. On day 15, a wound-healing rate of 97.46% was observed with SA@Ca^2+^/RCSPs nanofibers, whereas the rates 90.79%, 91.34% and 93.27% for control, PVP nanofibers and SA@Ca^2+^ nanofibers respectively. After 15 days, the wound-healing rates for these three groups reached more than 90%, but we do not know the internal wound situation, therefore we uses tissue biopsies to analyse the degree of wound healing of the four groups.

**Fig. 8:**
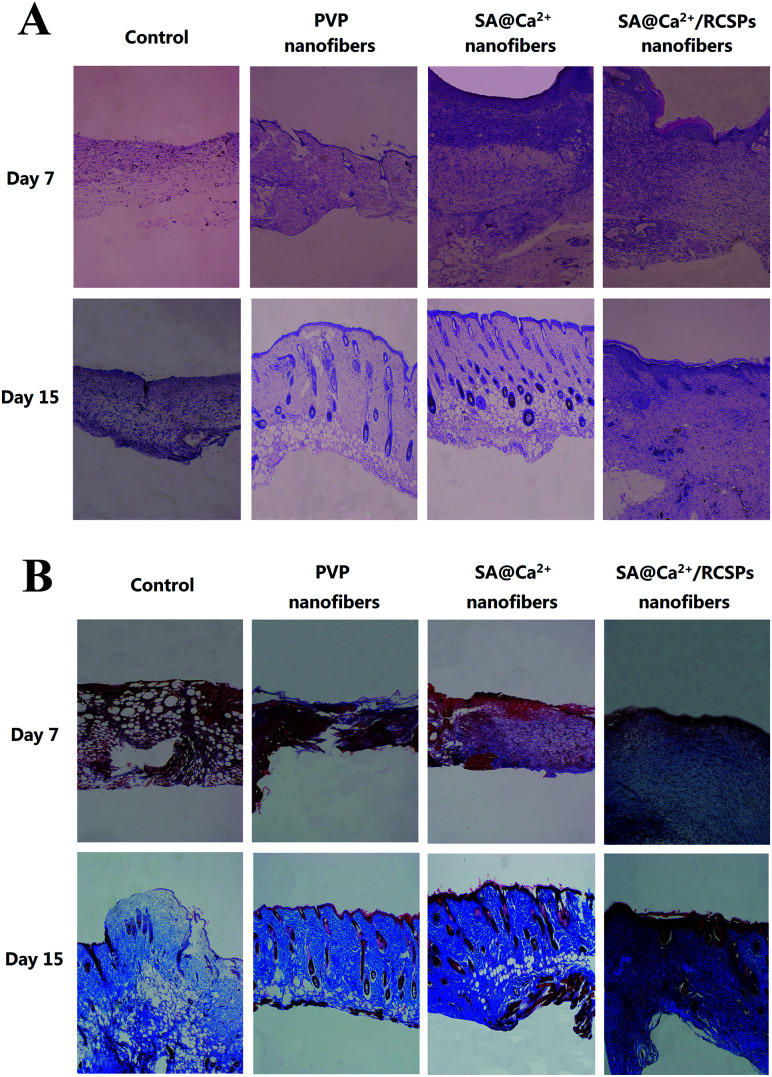
(A) HE-stained (Hematoxylin and Eosin-stained) of skin tissues treated with control, PVP nanofibers, SA@Ca^2+^ nanofibers and SA@Ca^2+^/RCSPs nanofibers. (B) Masson's trichrome staining of skin tissues treated with control, PVP nanofibers, SA@Ca^2+^ nanofibers and SA@Ca^2+^/RCSPs nanofibers. Magnification of the microscope = 100×.

The black mice from four groups were sacrificed on days 7 and 15 for tissue analysis. Fig. 8(A) shows the representative HE-stained of skin tissue of each group. On day 7, sparse granulation tissue from the wounds of control showed inflammatory cells and neutrophils, which indicate severe inflammation. PVP nanofiber-treated wounds showed inflammatory cells, neutrophils and small amounts of fibroblasts, and the granulation tissue at the wound has not grown yet. SA@Ca^2+^ nanofiber-treated wounds showed inflammatory cells, macrophages, neutrophils and fibroblasts. The fibroblasts of the upper layer were more intense. SA@Ca^2+^/RCSPs nanofiber-treated wounds showed small amounts of inflammatory cells, macrophages, neutrophils and fibroblasts, compared to the first three groups, and fibroblasts were not only dense but also uniform in distribution. On day 15, the control groups showed small amounts of inflammatory cells and increasing fibroblasts in the upper portion of the wound.

The other three groups showed epidermis and skin appendage regeneration, but the SA@Ca^2+^/RCSPs nanofiber-treated group compared with the other two groups displayed an epidermal layer of thick, skin appendage regeneration and the surrounding tissue to combine well, thick wave-like collagenous fiber. These findings indicate that the wound of the SA@Ca^2+^/RCSPs nanofiber-treated group was completely healed.

In parallel, Fig. 8(B) showed the four groups of representative tissue on days 7 and 15 using Masson trichrome staining. On day 7, collagen deposition occurred in the SA@Ca^2+^/RCSPs nanofiber-treated group, but not in the other three groups. On day 15, the SA@Ca^2+^/RCSPs nanofiber-treated group showed dense collagen deposition and thick wave-like collagenous fiber, compared to the other three groups. This observation was similar to the results of HE staining, indicating that the wound was completely healed.

The histological analysis showed that the SA@Ca^2+^/RCSPs nanofiber-treated group had the best therapeutic effect. When the composite nanofibers contact the wet wound, these molecules quickly become gels, which can stop bleeding and close the wound, and the RCSPs are rapidly released into the wound site to speed up cell growth and promote cell proliferation. Thus, granulation tissue or epithelium can gradually grow, thereby accelerating the healing time of the wound. In general, the carrier degradation of the reported most composite materials was slow and incomplete, resulting in drug release slow and incomplete, which makes drug waste. While according to the drug release model, RCSPs from the composite nanofibers in this work released rapidly and completely. And the healing time is much shorter than that reported for most composite materials.

## Conclusions

In the present study, utilizing the gelation reaction of calcium ions with alginate, SA@Ca^2+^/RCSPs nanofibers are prepared by coaxial electrospinning. Pore size analysis, gel test, contact angle test, TG-DTG, DSC, XRD and ATR-FTIR showed that solubility of the fiber mats was changed, producing a gelation reaction and good thermal properties. The liquid absorption of the composite nanofibers was consistent with the adsorption kinetics, and the release of RCSPs from the nanofibers was consistent with the release kinetics. In a short time, nanofiber mats can immediately stop bleeding and subsequently release drugs. *In vitro* wound healing studies showed that SA@Ca^2+^/RCSPs nanofibers increase wound healing rates and collagen deposition. The present study shows the feasibility of the gelation of SA@Ca^2+^/RCSPs composite nanofibers for wound healing. Moreover, according to the present work, we can further study the culture of skin fibroblasts *in vitro* and improve the functionalization of composite nanofibers, such as antibacterial activity, making the composite nanofibers more practical for the biomedical materials.

## Conflicts of interest

There are no conflicts to declare.

## Supplementary Material

RA-008-C8RA00784E-s001

RA-008-C8RA00784E-s002
