# Supplementary material for: Novel SA@Ca2+/RCSPs core–shell structure nanofibers by electrospinning for wound dressings
Source: RSC Adv. 2018 Apr 24;8(28):15558–66. doi: 10.1039/c8ra00784e (PMC9080105; doi:10.1039/c8ra00784e)
Supplement: RA-008-C8RA00784E-s001 [file RA-008-C8RA00784E-s001.pdf]

Electronic Supplementary Material (ESI) for RSC Advances.
This journal is © The Royal Society of Chemistry 2018

Electronic Supplementary Information

Prepared a novel SA@Ca2+/RCSPs core-shell structures nanofibers by electrospinning for wound dressings

Rui Li‡[a], Zhiqiang Cheng‡[a]*, Ruicheng Wen[b], Xiaodong Zhao, Xiaobin yu[a], Lin Sun[a], Yingying Zhang[a], Zhiyuan Han[c], Yafeng Yuan[a] and Lijuan Kang[a]*

[a] College of Resources and Environment, Jilin Agricultural University, Changchun 130118, People's Republic of China.

[b] College of Life Sciences, Jilin Agricultural University, Changchun 130118, People's Republic of China.

[c] College of Aerospace Engineering, Tsinghua University, Beijing, 100000, People's Republic of China.

* Corresponding author E-mail: czq5974@163.com (Zhiqiang Cheng)

‡ Zhiqiang Cheng and Rui Li contribute to the work equally.

Characterize the type and content of elements in the materials by Energy dispersive spectroscopy (EDS, SHIMADZU). Fig S1 show elements can be clearly observed, and the presence of Ca was proved in SA@Ca^{2+}/RCSPs nanofibers.

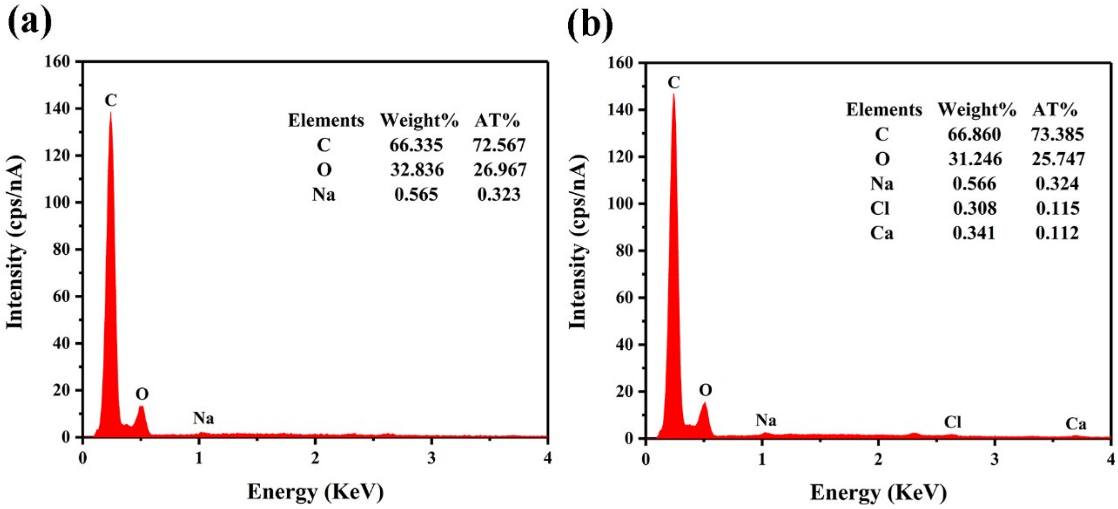

Figure S1. The EDS results of (a) SA@RCSPs nanofibers, (b) SA@Ca^{2+}/RCSPs nanofibers.
